# Economic Burden of Head and Neck Cancer Treatment in North India

**DOI:** 10.31557/APJCP.2019.20.2.403

**Published:** 2019

**Authors:** Akashdeep Singh Chauhan, Shankar Prinja, Sushmita Ghoshal, Roshan Verma

**Affiliations:** 1 *School of Public Health, *; 2 *Department of Radiotherapy, *; 3 *Department of Otolaryngology, Post Graduate Institute of Medical Education and Research, Chandigarh, India.*

**Keywords:** Head and neck cancer, radiotherapy, out of pocket expenditure, Catastrophic health expenditure

## Abstract

**Background:**

The rising cost of cancer treatment has imposed a huge financial burden on the affected households, leading to catastrophic outcomes and impoverishment. The present study was designed to estimate the economic burden incurred by households for the treatment of head and neck cancer (HNC) in India.

**Methods:**

The present study was undertaken in a large public sector tertiary care hospital of North India. A total of 159 patients were recruited at time of their first registration in the department of Radiation Oncology, and were followed after completion of their treatment. Another 288 were recruited within one month after completion of treatment. Economic burden was assessed in terms of out of pocket (OOP) expenditure incurred, prevalence of catastrophic health expenditure and distress financing (borrowing or selling of assets) related to different modalities of cancer treatment.

**Results:**

The average OOP expenditure incurred by a patient of HNC patient was INR 37, 845 (USD 563), which varied from INR 32,379 (USD 482) when a patient undergoes radiotherapy alone to INR 67,480 (USD 1,004) for surgery along with chemo-radiotherapy. Specifically, patients undergoing 2-DRT and IMRT alone had to spend INR 31,487 (USD 469) and INR 42,405 (USD 631) respectively. The prevalence of catastrophic health expenditure (CHE) and distress financing (DF) was 34% and 45% respectively. The odds of incurring both CHE and DF were found to be higher for patients in the lowest income quartile and for those in the younger age groups.

**Conclusion:**

Cancer imposes significant economic burden on households. The existing public health system should be strengthened to reduce OOP expenditure incurred by patients. In addition, enhanced coverage of risk pooling mechanisms should be ensured.

## Introduction

Cancer of the head and neck region (HNC) is the 7^th ^most common neoplasms worldwide, with around 70% of the total burden being contributed by developing nations. (Ferlay et al., 2015) South East Asia region (SEAR) contributes 32% (1.62 lakhs) of the incident HNC cases and 40% (1.13 lakhs) of the total mortality globally (Ferlay et al., 2015). Further, India is the home to around 3/4^th^ of SEAR burden of HNCs, both in terms of incidence (1.45 lakhs) and the mortality (1.05 lakhs). (Ferlay et al., 2015)

Treatment of cancer is a costly affair, as it requires intensive form of therapeutic techniques i.e., chemotherapy, radiotherapy and surgery, besides expensive diagnostics. (D’Cruz et al., 2012) Thus, provision of cancer care imposes a big financial liability for the payer - be it the State or household. In Indian context, much of this financial burden is borne by households as the Government contributes only around 29% of the health care expenditure and the rest is contributed by the households in the form of out of pocket (OOP) payments. (NHSRC, 2016; Bang et al., 2011).

Like other countries, India is committed to achieving Universal Health Coverage (UHC) (Ottersen and Norheim, 2014). Despite the acceptance of UHC as an important policy goal, 3.5% to 6.2% of the India’s population is pushed below the poverty line every year due to OOP expenses on treatment (Van Doorslaer et al., 2006; Bermam et al., 2010; Garg and Karan, 2009). Specifically, in relation to cancer, the odds of impoverishment and catastrophe due to a cancer treatment are 133% and 180% greater than the odds due to a communicable disease in India (Engelgau et al., 2012).

Besides direct medical expenditure on treatment, cancer imposes significant indirect costs. In India, around 70% of the cancer deaths occurred in people aged between 30–69 years (Dikshit et al., 2012). As a result, not only does cancer consume a substantial part of the family budget on treatment, but also indirectly impacts the economic well-being of households in the form of wage or income loss. 

There is limited evidence on OOP expenditure for cancer in India. Moreover, existing studies were undertaken almost a decade ago and none of these studies measured OOP expenditure specifically related to the HNCs (Mondal et al., 2014; Mohanti et al., 2011; Engelgau et al., 2012; Mahal et al., 2013) Considering this background, the present study was designed to estimate the OOP expenditure, financial risk (catastrophic health expenditure) and social impact (coping mechanisms) bear by the households getting treatment for HNC. 

## Materials and Methods

*Study Settings*

The present study was undertaken in the Department of Radiation Oncology of a tertiary care institute located in the North India. The institute caters to around 7 Indian states i.e., Punjab, Haryana, Himachal Pradesh, Jammu and Kashmir, Uttrakhand, Bihar, north-western part of Uttar Pradesh, and Union Territory of Chandigarh. A total of 6,171 and 1,020 cancer patients received outpatient consultation and inpatient care respectively, during 2015-16, in the radiotherapy department. 

**Table 1:** Socio-Demographic Characteristics of Head and Neck Cancer Patients

Characteristics/ Categories	N	%
Gender	403	90.2
Male		
Age		
Less than 30 years	23	5.1
30-40 years	42	9.4
40-50 years	79	17.7
50-60 years	151	33.8
Equal to and more than 60 years	152	34
Marital status		
Unmarried	21	4.7
Married	398	89
Others (divorcee/widow/widower)	28	6.2
Religion		
Hindu	383	85.7
Muslim	24	5.3
Sikh	40	8.9
Caste		
Scheduled castes and scheduled tribes	104	23.2
Other backward castes	121	27.1
General and others	222	49.6
Locality		
Rural	323	72.3
Education		
Illiterate	105	23.5
Primary	84	18.8
Middle	75	16.8
Matric	106	23.7
Senior secondary	36	8.1
Graduate and above	41	9.2
Income quartile		
Poorest (< INR 22,200)	112	25.1
Poor (INR 22,200 - INR 31,692)	112	25.1
Rich (INR 31,692 - INR 45,300)	112	25.1
Richest (> INR 45,300)	111	24.8
Presence of insurance/subsidy entitlement		
Yes	160	35.8
No	287	64.2
Treatment modality		
Radiotherapy alone	257	57.5
Radiotherapy along with chemotherapy	131	29.3
Surgery followed by radiotherapy	38	8.5
Surgery followed by radiotherapy and chemotherapy	21	4.7
Type of Radiotherapy		
Two dimensional radiotherapy	388	86.8
Intensity Modulated Radiotherapy	59	13.2
Stage of cancer		
I	18	4
II	37	8
III	82	18
IV	199	45
Missing	111	25
Total	447	100

**Table 2:** Out of Pocket (OOP) Expenditure During the Treatment of Head and Neck Cancer

Variable	Category	Direct medical OOP expenditure		P-value
		Mean in INR (SE)	Mean in USD (SE)	
Gender	Male	37,379 (1283)	556 (19)	0.245
	Female	42,114 (3681)	627 (55)	
Age	Less than 30 years	48,059 (4251)	715 (63)	0.001
	30-40 years	49,924 (5555)	743 (83)	
	40-50 years	39,902 (3295)	594 (49)	
	50-60 years	36,207 (1783)	539 (27)	
	Equal to and more than 60 years	33,521 (1867)	499 (28)	
Income quartile	Poorest	32,780 (1833)	488 (27)	0.007
	Poor	36,769 (2419)	547 (36)	
	Rich	37,411 (2386)	557 (36)	
	Richest	44,479 (2863)	662 (43)	
Locality	Rural	36,610 (1368)	545 (20)	0.1
	Urban	41,061 (2521)	611 (38)	
Presence of insurance/subsidy entitlement	Yes	35,287 (1879)	525 (28)	0.115
	No	39,272 (1568)	584 (23)	
Treatment modality	Radiotherapy alone	32,379 (1207)	482 (18)	<0.001
	Radiotherapy along with chemotherapy	37,192 (6065)	554 (90)	
	Surgery followed by radiotherapy	60,691 (1225)	903 (31)	
	Surgery followed by radiotherapy and chemotherapy	67,480 (4993)	1004 (121)	
Type of radiotherapy	Two dimensional radiotherapy	31,487 (1187)	469 (18)	0.013
	Intensity Modulated Radiotherapy	42,405 (4095)	631 (74)	
Stage	I	32,095 (4761)	478 (71)	0.35
	II	34,498 (2516)	513 (37)	
	III	33,149 (1946)	493 (29)	
	IV	37,878 (1840)	564 (27)	
Total		37,845 (1213)	563 (18)	

**Figure 1:**
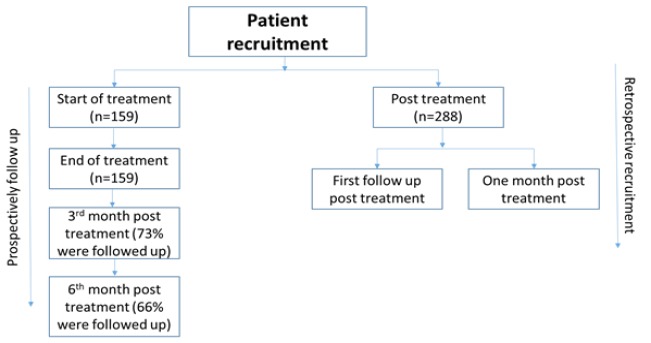
Flowchart Showing Recruitment of Patients

**Table 3:** Component Wise Breakdown of Out of Pocket (OOP) Expenditure on Different Treatment Modalities

Treatment modalities	Type of Radio-therapy	Direct medical expenditure in INR (SE)			Direct non-medical expenditure in INR (SE)		Total direct expenditure (SE)	
		Drugs	Diagnostic	Procedure/User fee	Travel	Boarding/Lodging/ Food	INR	USD
Radiotherapy alone	2DRT	4,007 (442)	4,838 (279)	3,797 (185)	12,868 (692)	5,967 (479)	31,487 (1225)	469 (18)
	IMRT	5,082 (1488)	6,618 (1322)	9,307 (1275)	12,535 (1679)	8,864 (1624)	42,405 (4993)	631 (74)
	Overall	4,095 (424)	4,984 (279)	4,247 (219)	12,840 (650)	6,212 (431)	32,379 (1208)	482 (18)
Radiotherapy along with chemotherapy	2DRT	7,485 (843)	5,715 (374)	3,862 (442)	13,589 (1299)	4,820 (482)	35,471 (2196)	528 (33)
	IMRT	8,836 (2113)	6,642 (866)	10,551 (1208)	13,316 (1961)	8,650 (1887)	47,995 (5188)	714 (77)
	Overall	7,670 (781)	5,842 (344)	4781 (461)	13,551 (1151)	5,346 (500)	37,192 (2052)	554 (90)
Surgery followed by radiotherapy	2DRT	14,751 (3756)	7,149 (1459)	10,750 (2654)	12,714 (1441)	7,231 (1537)	52,595 (7677)	783 (114)
	IMRT	24,569 (5177)	12,638 (4212)	16,412 (4720)	14,834 (3152)	7,809 (1684)	76,262 (8612)	1,135 (128)
	Overall	18,110 (3095)	9,027 (1749)	12,686 (2381)	13,439 (1420)	7,429 (1151)	60,691 (6065)	903 (31)
Surgery followed by radiotherapy and chemotherapy	2DRT	14,138 (5456)	5,657 (935)	12,800 (3551)	14,557 (1410)	10,721 (2836)	57,873 (8721)	861 (130)
	IMRT	31,757 (11538)	12,471 (3142)	19,171 (3302)	15,800 (2290)	7,493 (2395)	86,692 (15581)	1,290 (232)
	Overall	20,010 (5446)	7,929 (1372)	14923 (2647)	14971 (1184)	9,645 (2644)	67,480 (8152)	20,010 (5446)

*Treatment Process*

Patients suspected of HNC are first of all consulted and diagnosed in the outpatient clinic of the Otolaryngology (ENT) Department. After clinical investigations at this level, a cancer treatment plan for the patient is decided. The surgery is performed by the ENT surgeon of the Otolaryngology Department and for radiotherapy or chemotherapy, the patient is referred to the Department of Radiation Oncology/Radiotherapy.

*Study design*

The present study was based on mixed method design, in which 2 sets of patients were recruited. The first set of patients were recruited at the time of their registration in Radiotherapy Department and were prospectively followed up till 6 months of the end of their treatment (Figure 1). The second set consisted of those patients, who had completed their treatment and were retrospectively interviewed at the time of their first follow up or within one month post treatment (whichever was earlier). If the patient had undergone surgery, the expenditure incurred on the same was elicited retrospectively from both the groups.

Patients from both the groups were first of all contacted in the outpatient clinic (OPD) of the radiotherapy (RT) department. Those who were prospectively followed up, were recruited at the start of their treatment and were interviewed on a daily basis till the duration of their radiotherapy or chemo-radiotherapy (5-8 weeks), during which the patient along with accompanying care-taker (if any) visited the study hospital daily. These set of patients were also interviewed at 3rd and 6th month following treatment, either through telephonically or in the OPD clinic during their follow up visits. 

As per norms of the department, patients are required to come for their first follow up within 2 weeks post radiotherapy, but most of them make their first visit within a month post treatment. Thus, 2nd set of patients were recruited at the time of their first follow up or within one month post treatment. Payments receipts and bills were checked where available, to validate the reported expenditure. A sample size of 410 patients was estimated based on an average weekly OOP expenditure of INR 1062 on radiotherapy, standard deviation as 412 and INR 40 as level of precision at 95% confidence interval (Mohanti et al., 2011). 

*Data collection*

Patients were interviewed on their social demographic characteristics, household consumption expenditure, OOP expenditure on diagnosis/treatment and coping mechanisms for dealing with the same. If the patient had taken any treatment before coming to the study hospital, OOP expenditure on account of the same was also recorded. A pretested semi-structured schedule, adapted from previous studies done in the similar settings, was used to interview the patients (NSSO, 2006; Prinja et al., 2016; Prinja et al., 2018).

“Cost of Illness” approach (Rice, 2000) was followed to assess OOP expenditure, which classifies the same into direct and indirect expenditure. Expenditure on the diagnosis, drugs/consumables, hospitalization, user fee or procedure fee were considered as direct health care expenditure. Expenses for transportation, boarding, loading and food were taken as direct non health care expenditure. Wage/income loss by the patient and accompanying care-taker during the period of treatment was captured as indirect expenditure.

*Data Analysis*

Data was analysed using SPSS version 17 (SPSS Inc., Chicago, IL, USA). Mean OOP expenditure incurred on various treatment modalities, i.e., radiotherapy alone, chemo-radiotherapy and surgery followed by radiotherapy or chemo-radiotherapy was calculated. Further, expenditure on specific radiotherapy approach (Two dimensional radiotherapy and Intensity modulated radiotherapy) was also estimated. The OOP expenditure has been reported in Indian National Rupee (INR). For international comparison, expenses were converted into USD using a conversion rate of 1 INR to 67.19 USD for the year 2016-17, as reported by the World Bank.

Indirect expenditure was calculated for the duration of radiotherapy treatment using a human capital approach by assessing the wage loss of both the patients and carers (Lensberg et al., 2013). Financial risk was assessed in terms of catastrophic health expenditure and the distress ﬁnancing. Expenditure on cancer treatment which exceeded the threshold of 40% of non-food household consumption expenditure was considered as catastrophic health care expenditure (WHO, 2005; Moreno-Serra et al., 2011). Households which had either borrowed money (with or without interest) or had sold their assets (like land, home, cattle, etc.) to cope with the expenditure were classified to have faced distress financing (Huffman et al., 2011; Chauhan and Mukherjee, 2016; Prinja et al., 2016).

Multiple logistic regression analysis was performed to examine the risk of catastrophic health expenditure and the distress financing with covariate including age, sex, income status, treatment modality, insurance status, locality and stage at the time of diagnosis. Sensitivity analysis was carried out to assess the prevalence of catastrophic expenditure at varying cut off levels i.e., 20% to 50%.

*Ethical approval*

Ethical approval was obtained from the Institute Ethics Committee of the Post Graduate Institute of Medical Education and Research, Chandigarh, India (Reference number: NK/2490/Ph.D/6374). Written informed consent was obtained to interview the patients.

## Results

*Sample Characteristics*

Among 447 HNC patients recruited in the study, 90% were male, 68% were above 50 years of age, 86% belonged to Hindu religion, 3/4th resided in rural areas and 36% reported to have any form of subsidy entitlement/health insurance at the time of cancer treatment (Table 1). In terms of treatment undertaken, 57.5% had undergone radiotherapy alone, 29.3% undertook chemo-radiotherapy and the remaining 13% had surgery along with radiotherapy or chemo-radiotherapy. Specifically in relation to the radiotherapy, 87% underwent two dimensional radiotherapy (2-DRT) and the remaining had intensity modulated radiotherapy (IMRT) (Table 1). Excluding those who died, follow up rates were 73% and 66%, for those, who were followed up at 3rd and 6^th ^month post treatment respectively (Figure 1).

*Out of Pocket (OOP) Expenditure*

The average OOP expenditure incurred by a HNC patient was INR 37, 845 (USD 563), which varied from INR 32,379 (USD 482) to INR 67,480 (USD 1,004) depending upon the type of treatment undertaken (Table 2). Specifically, patients undergoing 2-DRT and IMRT alone had to spend an average amount of INR 31,487 (USD 469) and INR 42,405 (USD 631) respectively. Further, around 89% of the cases reported having incurred an average expenditure of INR 22,940 (USD 341) on treatment sought from other health providers before visiting the study hospital. 

The detailed component wise breakdown of OOP expenditure incurred on different treatment modalities has been shown in Table 3. When a patient undergoes radiotherapy alone, travel expenses constituted the major component of the overall expenditure, (2-DRT (41%) and IMRT (30%)). For patients who had undergone surgery (followed by radiotherapy or chemo-radiotherapy), expenses on drugs/consumables constituted the highest proportion (24%-37%). 

Indirect expenditure during the duration of radiotherapy was INR 18,588 (USD 277), which varied from INR 18,087 (USD 269) for those who had undergone 2-DRT to INR 22,300 (USD 331) for patients getting treatment on IMRT. Further, cumulative average OOP expenditure at 6 months post treatment was INR 4921 (USD 73). 

*Financial risk protection*

Among the recruited patients, 34% (n = 141) suffered from catastrophic health expenditure at 40% threshold. The prevalence of catastrophic expenditure changed to 68%, 48% and 22% when the threshold for catastrophic expenditure was taken as 20%, 30% and 50%, respectively. Multiple logistic regression at 40% threshold, showed that the odds of catastrophic expenditure was signiﬁcantly higher for those belonging to lower income quartile (OR: 5.6, 95% CI: 2.6–12.4, p-value: <0.001), as compared to the highest income quartile. (Supplementary material; Table 1) Secondly, those who got treatment with IMRT faced a higher odds of incurring catastrophic health expenditure (OR: 3.516; 95%CI: 1.61-7.66, p-value: <0.002) as compared to 2-DRT.

Forty five percent of the patients reported having faced distress financing. Like catastrophic expenditure, the risk of distress financing was more in the poor households as compared to the richer (OR: 3.4 95% CI: 1.6-7.1, p-value: 0.001) (Supplementary material; Table 2). Further, the odds of distress financing were 2.5 times higher in younger patients in the age group of 30-40 years (95% CI: 1.2-8.9, p-value: 0.022) as compared to older age group. Also, among treatment modalities, those who had undergone surgery had a higher odds of distress financing (OR: 10.2 95% CI: 2.6-35.6, p-value: 0.001) as compared to radiotherapy alone. Severity of the disease (stage of cancer), locality of the household and presence of any insurance/subsidy entitlement did not alter the risk of suffering from catastrophic expenditure and distress financing.

## Discussion

The rising cancer burden along with requirement of intensive form of therapeutic techniques and diagnostics for its treatment has led to increase in the cost of its care. In India, where around 65% of the overall health expenditure is paid out of pocket by the households, (NHSRC., 2016) the diagnosis of the cancer, could be devastating news for the families because of the constant financial drain caused by the nature of its treatment. The Government of India has introduced various publicly sponsored health insurance schemes across states, to reduce reliance on OOP expenditure. However, these schemes have not shown any reduction in the OOP payments (Prinja et al., 2017). 

We undertook this study, to estimate the OOP expenditure and catastrophic health expenditure associated with the treatment of HNC. We found that mean OOP expenditure incurred by a household having a patient of HNC was INR 32,379 (USD 482) for radiotherapy, INR 37,192 (USD 554) for chemo-radiotherapy and INR 67,480 (USD 1,004) for surgery followed by chemo-radiotherapy. The prevalence of catastrophic expenditure and the distress financing was 34% and 45% respectively. 

As most of the cancer treatment is available at the tertiary care level in India, the present study was undertaken in a large public sector tertiary care hospital located in the North India. A total of 447 patients of HNC were recruited, of which around 1/3rd of them were prospectively followed up and were interviewed on daily basis to minimize the recall bias. Remaining 2/3rd of the patients were interviewed following 1 month of the treatment. The methodology followed in various international (Living Standards Surveys) and national surveys (National Sample Survey), recommends a reference period of last 15-30 days for estimating OOP expenditure on out-patient consultation (OPD) and over the last 365 days for spending on hospitalization. (Kinnon et al.; 2005) Surgery requires hospitalization, while radiotherapy or chemo-radiotherapy, whether given on an OPD basis, are an intense and continuous form of treatment over a duration of 5-7 weeks. Hence, a recall period of the 1 month period was considered appropriate. We compared the mean OOP expenditure among those recruited prospectively with those who were interviewed retrospectively, and found that there was insignificant difference in the amount of OOP expenditure incurred between the 2 groups, suggesting absence of any systematic recall bias. 

One of the studies, (Mohanti et al., 2011) from a tertiary care hospital undertaken in 2006-07, reported an average OOP expenditure of INR 8184 (for a seven-week course of radiotherapy), which is around 1/4^th^ of the expenditure reported in our study. This study also reported that 59% of the expenditure on radiotherapy was spent on the transportation and food/lodging, which is also similar to distribution of cost in our study. The NSS 71st (Jan-June 2104) round, reported an average OOP expenditure of INR 24,526 in the public facilities (NSSO, 2015), which is double the expenditure reported in the similar survey in 2004-05 (Engelgau et al., 2012). 

The availability of cancer treatment in India is linked with inequities in accessibility to health care with uneven distribution of cancer care facilities across the country. (Gulia et al., 2017) This is also reflected through high proportion of overall OOP expenditure incurred on travel, boarding, lodging, etc. in the present study. The patients visiting the study hospital comes from more than 6 Indian states in the catchment area of radius varying between 300–350 kilometres. National Cancer Control Programme (NCCP), India, has recommended a proposal in the form of development of district cancer control societies and installation of cobalt radiotherapy machines at the level of district hospitals (DHs), thereby creating radiotherapy centres in each district. But the limited availability of radiation oncologists has become one of the deterring factor in the establishment of tele-therapy units at this level. But, this proposal can become feasible with the development of close networking and links between DHs and regional cancer centres, whereby radiotherapy planning in the form of CT simulation, contouring, dosimetry etc., can be done at the level of regional cancer centres under the direct supervision of radiation oncologist, whereas the administration of radiotherapy can be done at the level of DHs, which as per rule requires technical staff and not necessarily oncologists. 

OOP expenses are regressive in nature, i.e., poor families spends a higher percentage of their income on health care, as compare to the rich, as shown by studies on various chronic diseases (Ramachandran et al., 2007; Flores et al., 2008; Huffman et al., 2011; Chauhan and Mukherjee, 2016). Our findings on a similar lines showed that, although, the expenditure incurred by richer households were significantly higher than the poor households, but the poorest households spent around 55% of their consumption expenditure (CE) on cancer care as compared to richer households, who spent an equivalent of 25% of CE on treatment. Further, we found that OOP expenditure did not vary with the stage or severity of the disease, but it varied with type of treatment of treatment modality undertaken. The decision on the modality of treatment to be given to patient is not only based on the stage of the tumour, but also on the site of the tumour (based on clinical guidelines) and age and physical condition of the patient (considering those having better chance of prognosis or treatment outcome). In the present study, around 1/3rd of the younger patient (below 40 years of age) had undergone a treatment involving surgery or IMRT, i.e., an expensive treatment as compared to other modalities. Moreover, more than 50% of the patients of nasopharynx/paranasal/sinonasal/nasal cavity received IMRT.

A study, based on NSS, 2004-05 round, stated, that 50% of the households affected by cancer undertook borrowing and selling of assets to meet OOP expenditures, which is similar to our findings (Mahal et al., 2013). Further, the same study as well as the recent NSS round (Jan-Jun 2014), showed that those in the upper income groups show less dependency on borrowing or selling of assets as compared to low income ones, both in the urban and the rural areas, again a finding, similar to our study. 

*Limitations*

There were methodological limitations in assessment of indirect expenditure because of income/wage loss. Wage loss was assessed only during the duration of the treatment in the study hospital. As cancer is a chronic and debilitating disease, these wage losses could become a very significant part of the overall expenditure if calculated for the whole duration of illness. Moreover, a significant productivity loss occurs on account of premature death, which was not evaluated in our study.

In the present study, around 65% of the cancer patients were from the economically productive age group (below 60 years of age), of which 52% were daily wage labourers and cultivators. Accurately assessing indirect expenditure for those not in the formal sector becomes difficult, as these groups of people does not have any fixed employment and income. Further, human capital approach used in the present study, tends to create inequities while estimating indirect expenditure (Giled, 1996; Lensberg et al., 2013). As this approach is dependent upon the earnings generated and value of household productivity, it tends to assess high indirect costs for those in the high income groups and lower indirect costs for these in the lower income groups. Considering these drawbacks, we recommend undertaking future studies, based on robust methods, for accurately estimating the burden of indirect costs related to cancer. 

In conclusion, the present study showed that cancer affected households had to incur a signiﬁcantly high OOP on treatment in public sector hospital in India. As treatment in public sector hospitals is subsidized in India, the expenditure could be much higher in private sector hospitals, where treatment is totally funded by patients. Further, more than 1/3rd of the households fell in the trap of catastrophic health expenditure and distress financing. The study also highlights that poorest were hardest hit by the OOP payments, both in terms of catastrophic health expenditure and distress financing.

Our findings have important policy implications. Firstly, a high amount of OOP expenditure incurred on procedure, drugs and diagnostics, hints at strengthening the capacity of existing public health sector in terms of its infrastructure. Secondly, there is a need to increase the extent of ﬁnancial risk protection for cancer treatment. As, India moves on towards Universal Health coverage (UHC), high rates of catastrophic health expenditure on account of cancer treatment implies that there is a need to enhance coverage of risk pooling mechanisms for reducing reliance on OOP payments. Although, various publicly sponsored health insurance schemes have been launched across India, (Prinja et al., 2017) under which treatment of cancer is an integral component, but, there is a need to strictly revise the design and height of benefit package (the level of ﬁnancial protection as percentage of total health care costs) of these schemes, for adequately providing ﬁnancial risk protection. Finally, there is a need to focus on cancer prevention strategies in the form of screening programmes, for detection of cancer lesions in the early or pre-cancerous stage, and minimal radical treatment. 

*Funding Statement*

This research received no specific grant from any funding agency in the public, commercial, or not-for-profit sectors.
